# RAD51 paralogs promote homology-directed repair at diversifying immunoglobulin V regions

**DOI:** 10.1186/1471-2199-10-98

**Published:** 2009-10-28

**Authors:** Ellen C Ordinario, Munehisa Yabuki, Priya Handa, W Jason Cummings, Nancy Maizels

**Affiliations:** 1 Department of Biochemistry, University of Washington School of Medicine, Seattle, WA 98195-7650 USA; 2 Department of Immunology, University of Washington School of Medicine, Seattle, WA 98195-7650, USA; 3 Life Sciences Division, Lawrence Berkeley National Laboratory, University of California, Berkeley, CA, USA

## Abstract

**Background:**

Gene conversion depends upon the same factors that carry out more general process of homologous recombination, including homologous gene targeting and recombinational repair. Among these are the RAD51 paralogs, conserved factors related to the key recombination factor, RAD51. In chicken and other fowl, gene conversion (templated mutation) diversifies immunoglobulin variable region sequences. This allows gene conversion and recombinational repair to be studied using the chicken DT40 B cell line, which carries out constitutive gene conversion and provides a robust and physiological model for homology-directed repair in vertebrate cells.

**Results:**

We show that DT40 contains constitutive nuclear foci of the repair factors RAD51D and XRCC2, consistent with activated homologous recombination. Single-cell imaging of a DT40 derivative in which the rearranged and diversifying immunoglobulin λ_R _light chain gene is tagged with polymerized lactose operator, DT40 PolyLacO-λ_R_, showed that RAD51D and XRCC2 localize to the diversifying λ_R _gene. Colocalizations correlate both functionally and physically with active immunoglobulin gene conversion. Ectopic expression of either RAD51D or XRCC2 accelerated the clonal rate of gene conversion, and conversion tracts were significantly longer in RAD51D than XRCC2 transfectants.

**Conclusion:**

These results demonstrate direct functions of RAD51D and XRCC2 in immunoglobulin gene conversion, and also suggest that modulation of levels of repair factors may be a useful strategy to promote gene correction in other cell types.

## Background

**Figure 1:**
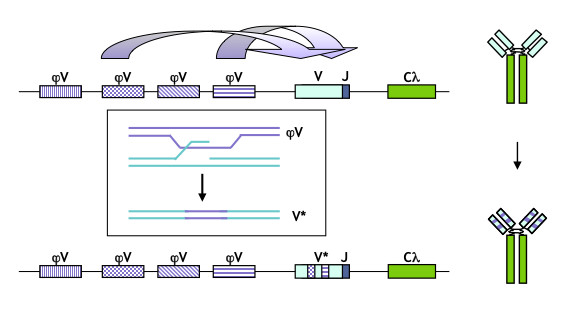
**Gene conversion diversifies chicken Ig genes**. Gene conversion at the chicken Igλ locus. The rearranged λ gene variable (VJ) and constant (C) region is transcribed to encode the Igλ light chain polypeptide (at right); upstream pseudo-V (ψV) donors are templates for sequence transfer (above). Tracts of templated mutation are evident in the diversified V region and the encoded protein (below). Gene conversion proceeds by a pathway in which the target V region is cleaved and then undergoes homology-directed repair templated by the ψV regions (boxed inset).

A diverse pre-immune immunoglobulin (Ig) repertoire is essential to vertebrate survival. In chickens and other fowl, the Ig heavy and light chain variable (V) regions are diversified by gene conversion, which transfers sequence information from upstream donor pseudo-V (ψV) regions to the rearranged and expressed heavy and light chain V regions (Figure 1) [1-8]. V region diversification in fowl occurs in a specialized organ, the bursa. The chicken B cell line, DT40, derives from a bursal lymphoma and constitutively diversifies both the heavy and light chain V regions by gene conversion [9]. DT40 also supports very high levels of homologous gene targeting, which has made it a valuable tool for genetic analysis of vertebrate cells as well as a powerful model for studying recombinational repair in a physiological context.

Ig gene conversion is initiated by the B cell-specific enzyme, activation-induced deaminase (AID) [10-13]. AID deaminates C to U in transcribed Ig genes, producing a U·G mismatch [14-17]; uracil-DNA glycosylase (UNG) removes U to produce an abasic site [18-21]; and the MRE11/RAD50/NBS1 (MRN) complex promotes gene conversion [22] using its abasic lyase activity to cleave at abasic sites [23].

Gene conversion and gene targeting are both impaired by deficiencies in factors involved in homology-directed repair, including MRE11 [24]; NBS1 [25,26]; the five RAD51 paralogs, RAD51B, RAD51C, RAD51D, XRCC2 and XRCC3 [27-30]; and BRCA1 and BRCA2 [30,31]. At the Ig genes, deficiencies of these factors, or deletion of [32] or repressive chromatin modifications at [33] the ψVλ donors does not simply diminish the clonal rate of gene conversion, but alters the mutational spectrum so that nontemplated mutations appear, analogous to those produced in somatic hypermutation in activated mammalian B cells.

To better understand the gene conversion pathway and how it may relate to other processes of recombinational repair, we have defined the localization and functions of RAD51D and XRCC2 in DT40 B cells. We find that RAD51D and XRCC2 form constitutive foci in normally proliferating DT40 cells. Single-cell imaging of DT40 PolyLacO-λ_R _cells, in which the rearranged and expressed λ_R _light chain gene can be visualized directly, showed that RAD51D and XRCC2 localize to the rearranged λ_R _allele. Colocalization reflects function in the diversification mechanism, as it is diminished upon expression of Ugi, which inhibits UNG activity; and correlates with enrichment at the rearranged λ_R _allele. In addition, ectopic expression of either RAD51D or XRCC2 accelerated the clonal rate of Ig gene conversion, and gene conversion tracts were significantly longer in RAD51D than XRCC2 transfectants. These results support a model in which RAD51D and XRCC2 participate directly in Ig gene conversion. They also support the notion that modulation of levels of repair factors may be useful for gene therapy strategies based on targeted gene correction.

## Results

### RAD51, RAD51D-GFP and XRCC2-GFP form nuclear foci in DT40 B cells

**Figure 2:**
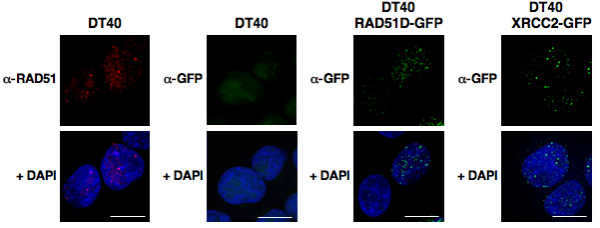
**RAD51, RAD51D-GFP and XRCC2-GFP form nuclear foci in DT40 B cells**. Representative immunofluorescent images of normally proliferating DT40 stained with anti-RAD51 (left) or anti-GFP antibodies (center left); and of DT40 RAD51D-GFP or DT40 XRCC2-GFP cells stained with anti-GFP antibodies (center right, right). Merged DAPI image, below; bar, 10 μm.

The chicken DT40 B cell line was derived from a bursal lymphoma and carries out constitutive diversification of its Ig genes by gene conversion [5-7]. The key homologous recombination factor, RAD51, has been shown to form constitutive nuclear foci in DT40 cells, which may reflect the recombinational activation characteristic of this cell line [28]. By staining with anti-RAD51 antibodies, we readily identified RAD51 foci in 31% of cells in an asynchronous culture (n = 241; e.g. Figure 2, left).

To determine whether RAD51D or XRCC2 form nuclear foci, we examined DT40 derivatives stably transfected with RAD51D-GFP or XRCC2-GFP, expressing C-terminal GFP-tagged proteins to ensure specificity. As the GFP-tagged proteins did not produce a sufficiently strong signal for direct imaging, we stained with anti-GFP antibodies to amplify the signals, and imaged cells by fluorescence microscopy. Staining with anti-GFP antibodies produced no background in untransfected DT40 cells (e.g. Figure 2, center left); but revealed constitutive punctate nuclear foci in normally proliferating DT40 RAD51D-GFP cells (e.g. Figure 2, center right; 36% of cells contained at least 3 foci; n > 200). A parallel analysis of DT40 XRCC2-GFP transfectants similarly revealed constitutive punctate nuclear foci in normally proliferating cells (e.g. Figure 2, right; 33% of cells contained at least 3 foci; n > 200).

### RAD51D-GFP and XRCC2-GFP localize to the rearranged λ_R _gene

**Figure 3:**
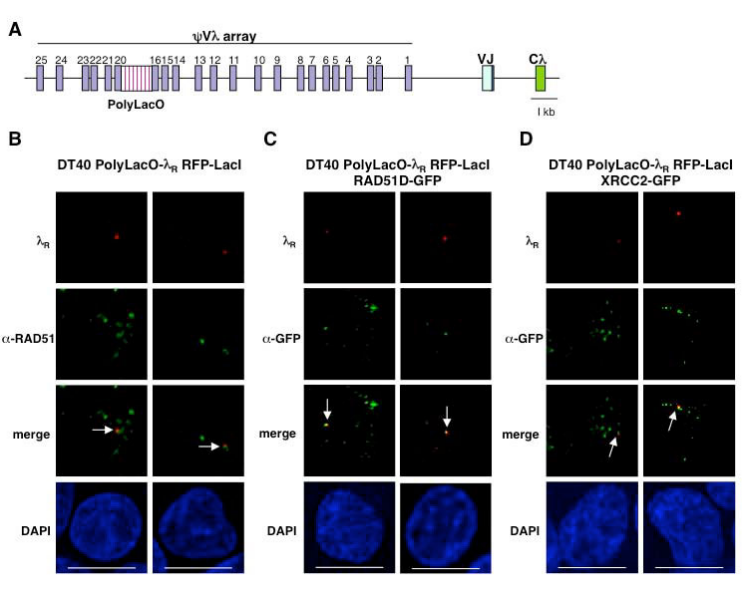
**RAD51, RAD51D-GFP and XRCC2-GFP foci localize to the diversifying λ_R _gene in DT40 PolyLacO-λ_R _cells**. (**A**) Igλ locus tagged with polymerized lactose operator (PolyLacO) in DT40 PolyLacO-λ_R_. The ψVλ array, PolyLacO, VJ and Cλ regions are indicated. (**B**) Immunofluorescent images of colocalizations of λ_R _gene with RAD51 in single DT40 PolyLacO-λ_R _RFP-LacI cells. Images of two representative cells are shown. λ_R_, red signal from RFP-LacI binding at Igλ_R_; α-RAD51, green; merge, λ_R _and RAD51 signal; DAPI, nuclear DNA. Bar, 10 μm. (**C**) Immunofluorescent images of colocalizations of the tagged λ_R _gene with RAD51D-GFP in stable DT40 PolyLacO-λ_R _RFP-LacI RAD51D-GFP transfectants. λ_R_, red signal from RFP-LacI binding at Igλ_R_; α-GFP, green; merge, λ_R _and GFP signal; DAPI, nuclear DNA. Bar, 10 μm. (**D**) Immunofluorescent images of colocalizations of the tagged λ_R _gene with XRCC2-GFP in stable DT40 PolyLacO-λ_R _RFP-LacI XRCC2-GFP transfectants. Notations as in panel C.

To further establish the significance of the RAD51, RAD51D and XRCC2 foci, we took advantage of a cell line recently developed by our laboratory, which can be used to directly image the rearranged and diversifying λ_R _allele in single B cells. In this line, DT40 PolyLacO-λ_R_, polymerized lactose operator (PolyLacO) is integrated within the ψVλ array just upstream of the rearranged and expressed λ_R _light chain gene (Figure 3A) [33,34]. Upon expression of red fluorescent protein fused to lactose repressor (RFP-LacI), RFP-LacI binds to PolyLacO to enable λ_R _to be imaged as a distinct red dot (e.g. Figure 3B, top). Localization of specific factors to the PolyLacO-tagged gene can be determined by calculating the fraction of cells in which the fluorescence signals of the gene and factor are overlapping (<0.2 μm spacing of the fluorescent peaks).

We assayed λ_R_/RAD51 colocalizations in asynchronous DT40 PolyLacO-λ_R _RFP-LacI cells, and observed colocalizations in 6.5% of cells (n = 167; e.g. Figure 3B, arrows). λ_R_/RAD51D-GFP colocalizations were observed in 15% of DT40 PolyLacO-λ_R _RFP-LacI RAD51D-GFP cells (n = 153; e.g. Figure 3C, arrows). λ_R_/XRCC2-GFP colocalizations were observed in 17.5% of DT40 PolyLacO-λ_R _RFP-LacI XRCC2-GFP cells (n = 434; e.g. Figure 3D, arrows).

**Figure 4:**
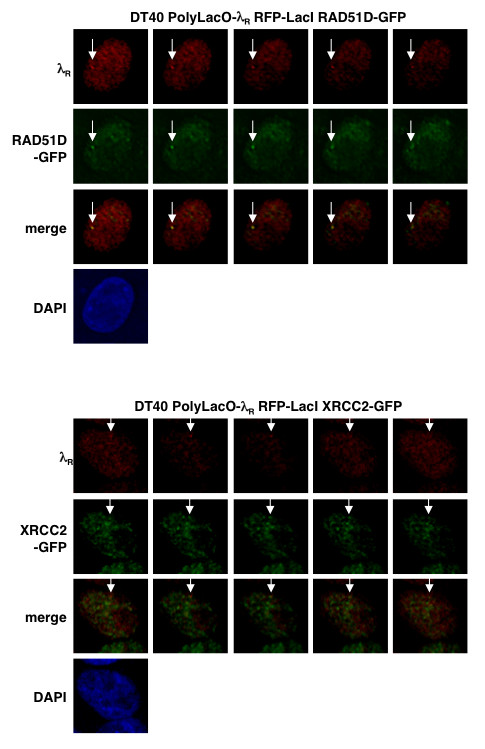
**Representative three-dimensional images of RAD51D-GFP and XRCC2-GFP colocalizations with the λ_R _gene**. Serial images of a single nucleus were taken in 0.2 μm sections, spanning a total field depth of 1.0 μm. Above, λ_R_/RAD51D-GFP colocalizations in stable DT40 PolyLacO-λ_R _RFP-LacI RAD51D-GFP transfectants. Below, λ_R_/XRCC2-GFP colocalizations in stable DT40 PolyLacO-λ_R _RFP-LacI XRCC2-GFP transfectants. λ_R_, red signal from RFP-LacI binding at Igλ_R_; RAD51D-GFP or XRCC2-GFP, green; merge, λ_R _and RAD51D-GFP or XRCC2-GFP signals; DAPI, nuclear DNA. Arrows indicate colocalizations.

To confirm that colocalization evident by confocal imaging reflected *bona fide *colocalization within the nucleus, we generated three-dimensional image stacks of RAD51D-GFP and XRCC2-GFP localizations at λ_R _in DT40 PolyLacO-λ_R _RFP-LacI cells. These "z-stacks" showed that colocalization was evident in multiple serial sections (Figure 4).

### λ_R_/RAD51D-GFP colocalization depends upon AID-initiated DNA damage

**Figure 5:**
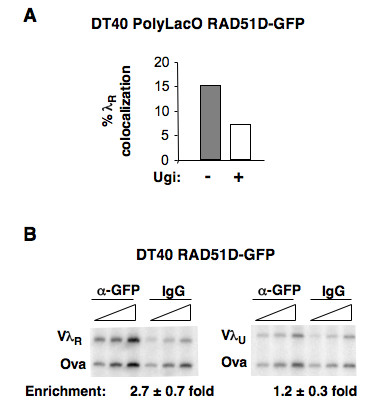
**λ_R_/RAD51D-GFP colocalization depends upon AID-initiated DNA damage and reflects enrichment of RAD51D-GFP at λ_R_**. (**A**) Comparison of the fraction of cells exhibiting λ_R_/RAD51D-GFP colocalizations in asynchronous cultures of DT40 PolyLacO-λ_R _RFP-LacI RAD51D-GFP cells expressing or not expressing Ugi. (**B**) Products of PCR amplification from fixed DT40 chromatin after immunoprecipitation with anti-GFP or polyspecific IgG (IgG) antibodies. Results shown are representative of results of three separate experiments, with two different chromatin preparations. Amplicons derived from the rearranged (Vλ_R_) or unrearranged (Vλ_U_) Vλ regions, or the ovalbumin gene (Ova). Amplification was carried out at two-fold dilutions of template (triangles). Enrichment was calculated as the ratio of the Vλ and Ova amplicons, relative to the IgG control; standard deviations shown.

Some background intersection of signals is inevitable in cells analyzed by confocal microscopy. To test whether colocalizations reflected function in DNA repair, we took advantage of the fact that the activity of UNG can be inhibited by expression of Ugi. UNG acts subsequent to AID to remove the uracil that results from cytidine deamination, creating an abasic site for attack by repair factors. UNG is essential in creation of the DNA break necessary to Ig gene conversion, and expression of Ugi has been shown to prevent Ig gene conversion in DT40 cells [20]. We generated stable DT40 PolyLacO-λ_R _RFP-LacI RAD51D-GFP Ugi transfectants, and compared colocalizations in these cells and in the parental DT40 PolyLacO-λ_R _RFP-LacI RAD51D-GFP cell line. We found that Ugi expression diminished the fraction of cells exhibiting λ_R_/RAD51D-GFP colocalizations from 15% to 7.0% (n = 199; Figure 5A). Thus, at least half of the λ_R_/RAD51D-GFP colocalizations reflect repair of lesions induced in the course of AID-initiated, UNG-dependent Ig gene diversification.

We have previously established the approximate level of background colocalizations in DT40 PolyLacO-λ_R _cells of two other factors essential for Ig gene diversification, Polη-GFP [35] and E2A [34]. Polη-GFP colocalizes with λ_R _in 11.4% of normally proliferating DT40 PolyLacO-λ_R _RFP-LacI Polη-GFP cells, but in only 5.4% DT40 PolyLacO-λ_R _RFP-LacI Polη-GFP Ugi transfectants [35]. As E2A acts upstream of AID, background for colocalizations of E2A was determined somewhat differently, by comparing localizations to the rearranged and unrearranged λ gene [34]. Colocalized λ_R_/E2A foci are evident in 26% of DT40 PolyLacO-λ_R _GFP-LacI cells, and in only 6.3% of DT40 PolyLacO-λ_U _GFP-LacI cells (in which the unrearranged λ_U _allele is tagged with PolyLacO). Thus, for the three factors analyzed thus far, RAD51D-GFP, Polη-GFP and E2A, background colocalization is in the range of 5.4 - 7.0%. By this criterion, the fraction of cells exhibiting λ_R_/RAD51 colocalizations is not significantly different from background, while the fraction of cells exhibiting colocalizations of RAD51D-GFP or XRCCC2-GFP is highly significant (*P *= 0.0001, χ^2 ^test).

### RAD51D-GFP is enriched at the rearranged but not unrearranged λ allele

To further establish that the observed colocalizations at λ_R _reflect events critical to the mechanism of Ig gene conversion, we compared physical association of RAD51D-GFP with the rearranged and diversifying or unrearranged and inactive λ alleles by chromatin immunoprecipitation (ChIP). Chromatin was immunoprecipitated from normally proliferating DT40 RAD51D-GFP cells with a polyclonal anti-GFP antibody or with nonspecific IgG control antibodies, and amplicons from either the rearranged or unrearranged Vλ region were amplified in duplex PCR reactions, along with a control amplicon, ovalbumin, as previously described [23,36]. The rearranged Vλ_R _region was 2.7-fold enriched relative to the ovalbumin control (Figure 5B, left), but there was no significant enrichment of the unrearranged Vλ_U _region (1.2-fold; Figure 5B, right). The level of enrichment was reproducible in three independent experiments. It is comparable to levels previously documented for association of RAD51D at breaks generated by I-SceI cleavage in mammalian cells [37]; and also in the range for association of other repair factors at target loci in eukaryotic cells, where interactions may be transient or occur only in only a small fraction of cells [23,36,38].

### RAD51D or XRCC2 expression accelerated Ig gene diversification in chicken B cells, but not human B cells

**Figure 6:**
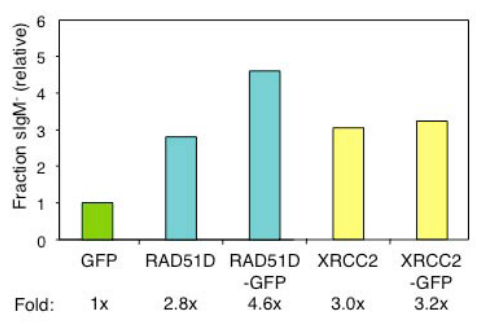
**RAD51D or XRCC2 expression accelerates the clonal rate of Ig gene diversification**. Fraction of sIgM loss variants (sIgM^- ^cells) in DT40 RAD51D, DT40 RAD51D-GFP, DT40 XRCC2 and DT40 XRCC2-GFP transfectants, normalized to DT40 GFP transfectants. Data are from two independent experiments; average fold increase is shown below.

To assay RAD51D and XRCC2 function in Ig gene diversification, we generated panels of stable, independent transfectants of a surface IgM-positive (sIgM^+^) subclone of DT40 expressing RAD51D-GFP, RAD51D, XRCC2-GFP, XRCC2, or a GFP control gene. Following clonal expansion, the diversification rate in each culture was determined using the sIgM loss assay [22,29,30,33], which quantitates the fraction of descendants of a single sIgM^+ ^cell which have become sIgM^- ^as a consequence of diversification. The sIgM loss assay detects acceleration or deceleration of the clonal diversification rate due not only to templated mutations, but also base substitutions, deletions and insertions that occur if the templated pathway has been impaired, as occurs if the balance of repair is disrupted, leading to mutagenesis rather than gene correction [29,30,32,33]. The fraction of sIgM^- ^cells was normalized to the mean fraction of loss variants in the control GFP transfectants (0.6%). This showed that the fraction of sIgM^- ^cells was 4.6-fold (*P *< 0.001, Mann-Whitney *U *test) and 2.8-fold (*P *= 0.096, Mann-Whitney *U *test) higher in the RAD51D-GFP and RAD51D transfectants, respectively, relative to the control (Figure 6). Similarly, the fraction of sIgM^- ^cells was 3.0-fold (*P *< 0.001, Mann-Whitney *U *test) and 3.2-fold (*P *= 0.099, Mann-Whitney *U *test) higher in the XRCC2-GFP and XRCC2 transfectants (Figure 6). Thus, expression of either RAD51D or XRCC2 accelerated Ig gene diversification. Acceleration could reflect an increase in the fraction of cells undergoing diversification, or acceleration of the reaction kinetics in individual cells.

To confirm that RAD51D or XRCC2 expression affects homology-directed repair but not other processes that diversify V region sequence, we asked if stable expression of RAD51D or XRCC2 affected clonal diversification in derivatives of the constitutively hypermutating human B cell line Ramos. The fraction of sIgM loss variants was almost identical in stable Ramos transfectants expressing RAD51D-GFP or XRCC2-GFP as in GFP control transfectants (*P *= 0.73 and 0.78, respectively, Mann-Whitney *U *test; data not shown). Thus ectopic expression of RAD51D and XRCC2 promotes homology-directed repair but does not influence somatic hypermutation, which is homology-independent.

### Ectopic expession of either RAD51D or XRCC2 promotes gene conversion

To ask whether ectopic RAD51D or XRCC2 expression promoted gene conversion or other mutagenic pathways, single sIgM^- ^cells of transfectants were isolated by flow cytometry, and Vλ regions were PCR-amplified and sequenced. After elimination of germline and duplicate sequences, sequences carrying unique mutations or combinations of mutations were aligned with the sequences of the ψVλ regions. Potential donors for each mutation were identified, if present in the ψVλ array, and the minimum homology that existed between potential ψVλ donors and recipient genes was determined for each sequence. Following the established convention [29,33]), changes from the germline sequence that shared a region of identity at least 9 bp in length with one or more ψVλ donors were identified as gene conversion events. Changes with no match, or which matched within a region < 9 bp in length, were identified as point mutations.

**Figure 7:**
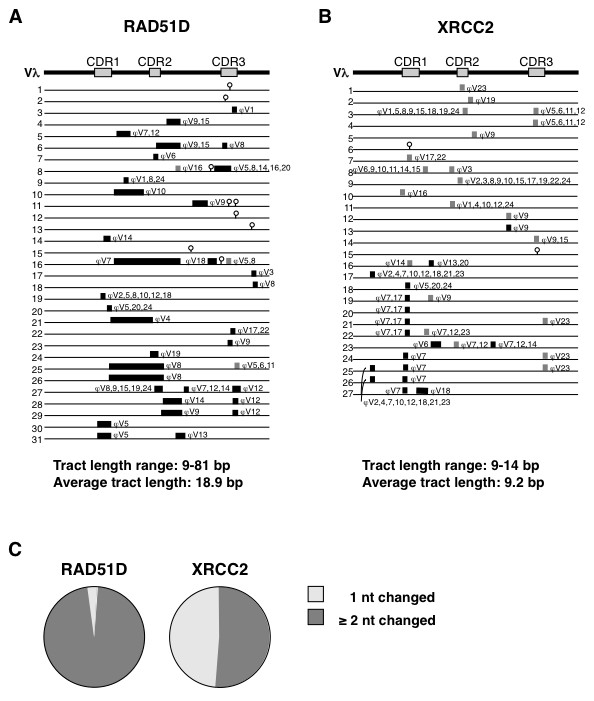
**Conversion tracts in DT40 RAD51D and XRCC2 transfectants**. (**A**) Schematic diagram of mutations in 31 Vλ regions from DT40 RAD51D-GFP transfectants, aligned with the germline Vλ region (top). Complementarity-determining regions (CDRs) which make major contacts with antigen are indicated. Each Vλ sequence is represented as a horizontal line; bars identify gene conversion tracts (black, 2 or more nt differences with germline; gray, one nt difference); and lollipops identify point mutations with no match in the germline sequence. Potential ψV donors for each converted tract are identified above the bars. Range and average length of conversion tracts indicated below. (**B**) Schematic diagram of mutations in 27 Vλ regions from DT40 XRCC2-GFP transfectants, aligned with the germline Vλ region (top line). Notations as in panel A. (**C**) Pie chart showing relative fractions of gene conversion events resulting in changes at 1 nt or 2 or more nt, in Vλ genes from DT40 RAD51D-GFP and DT40 XRCC2-GFP transfectants.

From 21 independently transfected lines expressing RAD51D, sequences of 171 Vλ regions were determined, and 31 unique mutated sequences further analyzed (Figure 7A; Additional File 1). This analysis showed that these sequences contained a total of 148 single base substitutions, insertions or deletions, 138 of which were due to gene conversion. Thus, in RAD51D transfectants, 93% of mutations match sequences in ψVλ donors.

A similar analysis was carried out on 19 independently transfected lines expressing XRCC2, from which sequences of 162 Vλ regions were determined, and 27 unique sequences further analyzed. These harbored a total of 77 single base changes, 75 of which matched sequences of ψVλ donors, and thus appeared to result from gene conversion (Figure 7B; Additional File 2). Thus, in XRCC2 transfectants, 97% of sequence changes match sequences in ψVλ donors.

### RAD51D expression increases gene conversion tract lengths

Gene conversion repair tract lengths can be estimated by determining the boundaries of homology between each tract recipient and its possible ψVλ donors. These homologies were determined for the DT40 RAD51D and DT40 XRCC2 transfectants. A mutation was scored as templated only if there was homology to a ψVλ donor within a window at least 9 nt in length, so 9 nt was the lower limit on tract length. Homologies ranged from 9-81 bp in Vλ regions of RAD51D transfectants (Figure 7A), and from 9-14 bp in Vλ regions of XRCC2 transfectants (Figure 7B). The average minimum repair tract length was thereby estimated to be 18.9 bp in RAD51D transfectants; and 9.2 bp in XRCC2 transfectants. This difference is highly significant (*P *< 0.001, Mann-Whitney *U *test). The average minimum tract length in control GFP transfectants was 9.9 bp (Additional File 3), significantly different from RAD51D transfectants (*P *= 0.012, Mann-Whitney *U *test) but comparable to XRCC2 transfectants (*P *= 0.11, Mann-Whitney *U *test). Thus, RAD51D expression resulted in longer tract lengths.

We also distinguished the fraction of templated events that contained two or more templated base changes, rather than a single base change. In the DT40 RAD51D transfectants, a minimum of 36 gene conversion events could account for all the templated changes in the sequenced Vλ regions, 33 (92%) of which caused changes at two or more nt; and three that caused a change at a single nt (Figure 7C). (This latter category could in principle result from a nontemplated mutation that coincidentally matched germline sequence.) In the DT40 XRCC2 transfectants, a minimum of 38 gene conversion events could account for all mutation tracts, 17 (45%) resulting in ≥ 2 changes and 21 (55%) resulting in a single nt change (Figure 7C). Thus, the fraction of gene conversion events producing ≥ 2 templated base differences was strikingly different between RAD51D and XRCC2 transfectants (*P *< 0.0001, Fisher's exact test).

## Discussion

The five RAD51 paralogs have previously been shown to be necessary for Ig gene conversion in experiments demonstrating that ablation of any of these genes causes AID-initiated mutagenesis to switch from a templated to a nontemplated repair pathway [27-30]. We have presented several kinds of evidence consistent with direct function of both RAD51D and XRCC2 in Ig gene conversion. Imaging colocalizations with λ_R _provided one snapshot of events in Ig gene conversion. We documented λ_R_/RAD51D-GFP colocalizations in 15% of cells, and λ_R_/XRCC2-GFP colocalizations in 17.5% of cells, both significantly greater than background. Colocalizations were shown to correlate both functionally and physically with active Ig gene conversion. In addition, ectopic expression of either RAD51D or XRCC2 accelerated Ig gene conversion in chicken DT40 B cells, but did not affect the rate of diversification in human Ramos B cells, which depend upon low fidelity polymerases and not homologous recombination to repair DNA damage initiated by the activity of AID. Notably, expression of RAD51D but not XRCC2 increased the conversion tract length.

DT40 cells are unusual in that they contain constitutive foci of repair factors, including the key recombination factor, RAD51 [28] as well as the RAD51D and XRCC2 foci that we have documented. These constitutive foci probably reflect the recombinationally active state of DT40 B cells, which support ongoing Ig gene conversion and very efficient homologous gene targeting. While it is not possible to confirm that gene conversion is ongoing at a specific λ_R _allele imaged in a single cell, expression of Ugi, which inhibits gene conversion, caused a 50% reduction in the fraction of cells exhibiting λ_R_/RAD51D-GFP colocalizations. This functional analysis suggests that about half the colocalizations observed are at sites of active gene conversion. Other colocalizations may reflect background inherent to confocal microscopy, a possibility consistent with evidence that expression of Ugi similarly reduced λ_R_/Polη-GFP colocalizations by approximately 50% [35]. Participation in the gene conversion mechanism was further supported by establishing that RAD51D-GFP is specifically enriched at the rearranged λ_R _allele, which undergoes diversification; and not at the inactive, unrearranged λ_U _allele.

The fraction of cells in which colocalizations are evident will be determined both by the fraction of cells in which colocalizations occur and by the duration of colocalization. λ_R_/RAD51 colocalizations were evident in only 6.5% of cells, not significantly greater than background. The absence of a significant fraction of cells exhibiting λ_R_/RAD51 colocalizations could mean that RAD51 associations with the diversifying Ig genes are relatively transient; alternatively, Ig gene conversion may represent a specialized pathway in which recombination does not depend upon RAD51.

### Accelerated gene conversion and increased tract length in DT40 RAD51D transfectants

Expression of either RAD51D or XRCC2 caused comparable 3-fold acceleration in the rate of diversification, as measured by the sIgM loss assay. Distinct mechanisms could account for this increase. Expression of these factors may cause a greater fraction of cells to carry out productive diversification, or alter the reaction kinetics of diversification in individual cells.

Gene conversion tracts were significantly longer in DT40 RAD51D than DT40 XRCC2 transfectants. In the latter cell lines tract length was identical to that in control DT40 GFP transfectants. Gene conversion tract length is almost certainly closely regulated *in vivo*, as tract length has clear biological consequences. Short conversion tracts may be advantageous at diversifying Ig genes as they enable the recipient gene to accumulate a patchwork of mutations from multiple different donors, which contributes diversity to the repertoire. Short conversion tracts will also cause modulated rather than drastic changes in antibody specificity. In contrast, longer conversion tracts would tend to create greater diversity in the same number of rounds of diversification; but would overwrite not only germline sequence but also sequence from previous rounds of gene conversion, in effect erasing mutations.

### Possible implications for gene correction strategies

There is considerable interest in the possibility of correcting mutations associated with genetic disease by gene correction [39]. In this approach, a DNA break is targeted at or near a defective gene, and ensuing DNA repair uses a homologous donor to correct the genetic defect, thereby restoring gene function. Strategies for elevating the efficiency of gene correction have focused on each of the steps in this pathway. Efforts to design nucleases that create specific breaks have met with encouraging recent success [40-43]. A critical limitation is the relatively low efficiency of homology-directed repair in most vertebrate cell types. In a few cases it has been shown that homology-directed repair, or the related process of homologous gene targeting, can be enhanced by increasing or diminishing levels of specific repair factors [44-49]. This has suggested that systematic analysis of the ability of repair factors to stimulate homology-directed repair might identify useful strategies to promote targeted gene correction. Our results identify RAD51D and XRCC2 as potential candidates for such approaches.

It is not possible to know whether a factor that promotes gene conversion at the Ig genes in chicken B lymphocytes would have a similar function in other cell types or other species. Nonetheless, by establishing that ectopic expression of repair factors can enhance homologous recombination in this context, our results provide proof in principle for the likely utility of extending this approach to other targets and other cell types. The ability of RAD51D expression to augment repair tract length is of particular potential utility for application to targeted gene correction. In targeted gene correction, repair tract length in effect determines how near a target mutation nuclease cleavage can occur and still promote useful repair. Thus, longer tracts are predicted to be advantageous in this context. The evidence that ectopic expression of RAD51D enhances both the clonal efficiency of gene conversion and repair tract length suggests the utility of considering both these parameters in future efforts to promote gene correction.

## Conclusion

DT40 contains constitutive nuclear foci of the repair factors RAD51D and XRCC2, consistent with activated homologous recombination. Single-cell imaging of DT40 PolyLacO-λ_R _cells showed that RAD51D and XRCC2 localize to the diversifying λ_R _gene. Colocalization correlates with function in diversification, and with physical association with the rearranged λ_R _allele. Ectopic expression of either RAD51D or XRCC2 accelerated the clonal rate of gene conversion, and conversion tracts were significantly longer in RAD51D than XRCC2 transfectants. These results demonstrate direct functions of RAD51D and XRCC2 in immunoglobulin gene conversion, and also suggest that modulation of levels of repair factors may be a useful strategy for gene correction in other cell types.

## Methods

### Plasmid constructs

RAD51D and XRCC2 cDNAs were isolated from the human B cell line, Raji, as mammalian RAD51 paralogs are known to function in chicken cells [28]. Following reverse transcription with the ProtoScript first strand cDNA synthesis kit (New England BioLabs, Ipswich, MA), RAD51D cDNA was amplified with the forward primer 5'-TAAGATCTACCATGGGCGTGCTCAGGGTC-3' and the reverse primers 5'-ATACCGGTGGTCATGTCTGATCACCCTG-3' or 5'-ATACCGGTGGTGTCTGATCACCCTGTAA-3', which carries an in-frame stop codon. PCR products were cloned into the pCR2.1-TOPO vector (Invitrogen, Carlsbad, CA), excised with BglII and AgeI, and subcloned into the pEGFP-N1 vector (Clontech, Mountain View, CA) to generate pRAD51D-GFP and pRAD51D, respectively. XRCC2 cDNA was similarly amplified with the forward primer 5'-CACCATGTGTAGTGCCTTCCATAGGGCTGAGTCT-3' and the reverse primer 5'-TCAACAAAATTCAACCCCACTTTCTCC-3' containing an in-frame stop codon and cloned into the pcDNA3.1/V5-His-D-TOPO vector (Invitrogen, Carlsbad, CA) to generate pXRCC2; or cDNA amplified with the primers 5'-AAAAAGGTACCGATGTGTAGTGCCTTCCATAGGGC-3' and 5'AAAAAACCGGTCCAC-AAAATTCAACCCCACTTTCTC-3', excised with KpnI and AgeI, and cloned into the pEGFP-N1 vector to generate pXRCC2-GFP. The Ugi expression vector [18] was provided by Dr. Michael Neuberger (Medical Research Council Laboratory of Molecular Biology, Cambridge, UK).

### Cell culture, transfection and analysis

Cell culture, transfection and cell cycle analysis were carried out as described [22,34]. Control experiments confirmed that cell proliferation and cell cycle distribution were unaltered in all stable transfectants prior to further analysis. Western blotting with antibodies against the tag was performed to verify comparable levels of expression of C-terminal tagged RAD51D or XRCC2 in stable transfectants (not shown). Uracil glycosylase activity was assayed [23] to verify Ugi expression.

### Immunofluorescence microscopy

Cells (~3 × 10^5^) were deposited onto glass slides using Shandon Cytospin3 (800 rpm, 4 min; Thermo Fisher Scientific, Waltham, MA), fixed with 2% paraformaldehyde for 20 min, permeabilized with 0.5% NP-40 for 15 min, and stained as previously described [22,34,50]. Primary antibodies for staining were monoclonal anti-GFP (3E6, 1:100; Molecular Probes, Eugene, OR) and polyclonal anti-RAD51 (1:500; provided by Dr. Charles Radding, Yale University, New Haven, CT). Secondary antibodies (Molecular Probes, Eugene, OR) were: for anti-GFP, Alexa Fluor 488-conjugated anti-mouse IgG (1:1500); and for anti-RAD51, Alexa Fluor 488- and 594-conjugated anti-rabbit IgG (1:1500). Specific recognition of chicken RAD51 was verified by immunoblotting (data not shown). Stained cells were visualized using Leica SP1 confocal (Leica Microsystems, Bannockburn, IL) and DeltaVision deconvolution (Applied Precision, Issaquah, WA) microscopes with 60× and 100× objectives, and images were processed using Leica LCS (Leica Microsystems, Bannockburn, IL) and softWoRx (Applied Precision, Issaquah, WA) software.

To image PolyLacO, DT40 PolyLacO-λ_R _cells were stably transfected with an RFP-LacI expression construct (a derivative of p3'SS-GFP-LacI [51,52] in which GFP was replaced with DsRed-monomer (Clontech, Mountain View, CA), and visualized as above. Cells that contained RAD51, RAD51D-GFP or XRCC2-GFP foci superimposed with RFP-LacI foci were considered positive for colocalization. Cross sectional images measuring 0.2 μm apart were analyzed with a line profile tool of the image software to confirm colocalization occurred within the same focal plane. Fluorescent signals were sometimes partially rather than completely overlapping, which may reflect the considerable distance (~17 kb) between the PolyLacO-tag and the Vλ region; both configurations were scored as localization. Significance of colocalization was analyzed with the Pearson's χ^2 ^test.

### Chromatin immunoprecipitation

Chromatin preparation and immunoprecipitation (IP) were performed as described previously [23,36]. Anti-GFP antibody was purchased from Abcam (Cambridge, MA). Amplifications were performed using Fast-Start Taq polymerase (Roche, Indianapolis, IN) and the following oligonucleotide primers: for the DT40 rearranged Vλ region, 5'-GCCGTCACTGATTGCCGTTTTCTCCCCTC-3' and 5'-CGAGACGAGGTCAGCGACTCACCTAGGAC-3'; for the unrearranged Vλ, 5'-CAGGAATGGAGGTGGGACT-3' and 5'-GCCGTCACTGATTGCCGTTTTCTCCCCTC-3' (one of the rearranged Vλ region oligos); for ovalbumin, 5'-ATTGCGCATTGTTATCCACA-3' and 5'-TAAGCCCTGCCAGTTCTCAT-3'. PCR products were quantified using ImageQuant software (Amersham, Piscataway, NJ). Enrichment was calculated as the ratio of the Vλ amplicon to the ovalbumin amplicon, normalized to the ratio of products from IP with polyspecific IgG antibodies: Enrichment Vλ = [anti-GFP (Vλ/ovalbumin)]/[IgG (Vλ/ovalbumin)]. Enrichment was compared at three template concentrations, to confirm that assays were within the linear range of PCR. Results shown are representative of three amplifications from two independent chromatin preparations.

### Ig diversification rates and Vλ_R _sequence analysis

The sIgM loss assay was used to quantitate Ig gene diversification [22,29,30,33]. Briefly, independent transfectants (typically 30-65 clones) were cultured for 4-6 wk posttransfection, stained with monoclonal anti-chicken IgM conjugated to RPE (1:500; SouthernBiotech, Birmingham, AL), and analyzed by flow cytometry. The percentage of sIgM^- ^cells was calculated as the ratio of the number of cells with 8-fold or greater decrease in RPE intensity to the RPE of the sIgM^+ ^population [29,30]. Statistical analyses used the R software package , and sIgM loss levels in transfectants were compared to vector controls using the Mann-Whitney *U *test.

Rearranged Vλ regions were amplified from flow-sorted single sIgM^- ^cells and sequenced as described [22,33]. Sequence alignment was done with the Sequencher program (Gene Codes Corporation, Ann Arbor, MI). A mutation in Vλ was designated as templated if one or more base substitutions within a 9 bp tract exactly matched to a ψVλ region; and otherwise as nontemplated [29]. Gene conversion tract lengths and mutational spectra of RAD51D and XRCC2 transfectants were compared using the Mann-Whitney *U *test and Fisher's exact test, respectively.

## Authors' contributions

ECO participated in construction of cell lines and immunofluorescence microscopy, analyzed diversified V region sequences, and drafted the manuscript. MY participated in construction of cell lines and immunofluorescence microscopy. PH participated in analysis of Ig diversification rates. WJC carried out chromatin immunoprecipitation analyses. NM conceived of the study, and participated in its design and coordination. All authors read and approved the final manuscript.

## Supplementary Material

Additional file 1 **Vλ sequences in DT40 RAD51D-GFP transfectants**. Sequences of 31 diversified Vλ sequences, aligned to the Vλ germ line sequence, with CDRs underlined. Blue, gene conversion tracts; red, nontemplated mutations. Click here for file

Additional file 2 **Vλ sequences in DT40 XRCC2-GFP transfectants**. Sequences of 27 diversified Vλ sequences, aligned to the Vλ germ line sequence, with CDRs underlined. Blue, gene conversion tracts; red, nontemplated mutations. Click here for file

Additional file 3 **Conversion tracts in DT40 GFP transfectants**. (**A**) Sequences of 12 diversified Vλ sequences, aligned to the Vλ germ line sequence, with CDRs underlined. Blue, gene conversion tracts; red, nontemplated mutations. (**B**) Schematic diagram of mutations in 12 Vλ regions from DT40 GFP transfectants, aligned with the germline Vλ region (top line). Notations as in Figure 7A. Click here for file
