# Supplementary figures and images for: RAD51 paralogs promote homology-directed repair at diversifying immunoglobulin V regions
Source: BMC Mol Biol. 2009 Oct 28;10:98. doi: 10.1186/1471-2199-10-98 (PMC2774322; doi:10.1186/1471-2199-10-98)

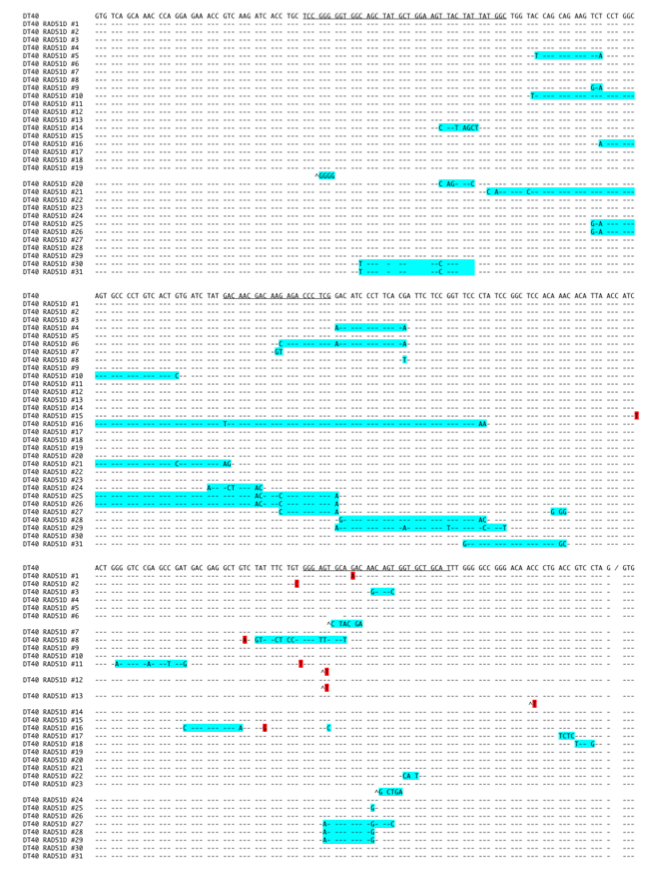

Supplement: Additional file 1 — Vλ sequences in DT40 RAD51D-GFP transfectants. Sequences of 31 diversified Vλ sequences, aligned to the Vλ germ line sequence, with CDRs underlined. Blue, gene conversion tracts; red, nontemplated mutations. [file 1471-2199-10-98-S1.TIFF]

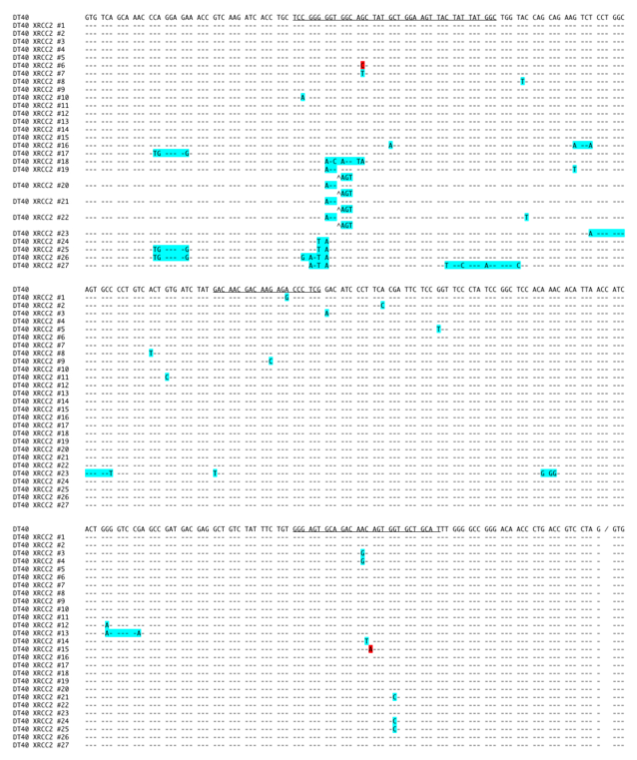

Supplement: Additional file 2 — Vλ sequences in DT40 XRCC2-GFP transfectants. Sequences of 27 diversified Vλ sequences, aligned to the Vλ germ line sequence, with CDRs underlined. Blue, gene conversion tracts; red, nontemplated mutations. [file 1471-2199-10-98-S2.TIFF]

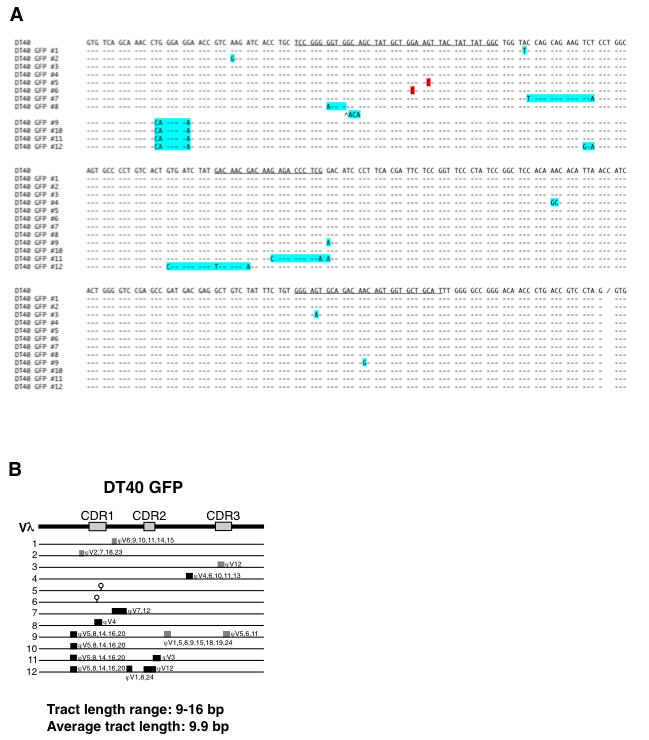

Supplement: Additional file 3 — Conversion tracts in DT40 GFP transfectants. (A) Sequences of 12 diversified Vλ sequences, aligned to the Vλ germ line sequence, with CDRs underlined. Blue, gene conversion tracts; red, nontemplated mutations. (B) Schematic diagram of mutations in 12 Vλ regions from DT40 GFP transfectants, aligned with the germline Vλ region (top line). Notations as in Figure 7A. [file 1471-2199-10-98-S3.TIFF]
